# Breast metastasis from squamous cell carcinoma of the oropharynx: a case report

**DOI:** 10.1186/s13256-017-1500-3

**Published:** 2017-12-22

**Authors:** Raffaele Longo, Emmanuelle Melgar, Marco Campitiello, Francesca Plastino, Nada Eid, Isabelle Quirin, Laurent Hennequin, Yves Grignon, Michel Gunther, Philippe Quétin

**Affiliations:** 1 Division of Medical Oncology, Centre Hospitalier Régional (CHR) Metz-Thionville, 1 Allée du Château, 57085 Ars-Laquenexy, France; 2 Division of Radiology, Centre Hospitalier Régional (CHR) Metz-Thionville, 1 Allée du Château, 57085 Ars-Laquenexy, France; 3 Division of Pathology, Centre Hospitalier Régional (CHR) Metz-Thionville, 1 Allée du Château, 57085 Ars-Laquenexy, France; 4 Division of Gynecology, Centre Hospitalier Régional (CHR) Metz-Thionville, 1 Allée du Château, 57085 Ars-Laquenexy, France; 5 Division of Radiotherapy, Centre Hospitalier Régional (CHR) Metz-Thionville, 1 Allée du Château, 57085 Ars-Laquenexy, France

**Keywords:** Metastasis, Head and neck, Breast, Oropharynx

## Abstract

**Background:**

Breast metastases from extramammary tumors are extremely rare, the most common primary tumors being contralateral breast carcinoma, followed by lung, gynecological, gastrointestinal, melanoma, and hematological cancers. Only a few cases deriving from head and neck squamous cell carcinoma have been reported in the literature to date.

**Case presentation:**

We report a case of a 47-year-old Caucasian woman who presented to our hospital with a solitary breast lesion in the right upper external quadrant associated with multiple bone and visceral metastases. Two years before, she had undergone radical resection of a squamous cell carcinoma of the oropharynx (stage pT2, pN1), which was followed by adjuvant radiotherapy. Breast ultrasound showed a hypoechogenic tumor lesion of 4 cm in the right upper external quadrant that was associated with multiple axillary and infra-/supraclavicular adenopathies. A positron emission tomographic scan documented multiple visceral and bone metastases with a single hypermetabolic lesion of the right breast. The results of histology and immunohistochemistry were consistent with a metastasis from a squamous cell carcinoma. The patient died of acute respiratory insufficiency 1 month after her breast metastasis diagnosis and before starting any systemic antitumoral treatment.

**Conclusions:**

Although breast metastases are extremely rare, they should be considered in any patient with a history of cancer and confirmed by histology and immunohistochemistry because they are very difficult to distinguish from other primary breast tumors based only on clinical and radiological features. There are no standardized treatment guidelines for breast metastasis management. Surgery and radiotherapy can play a role in symptom palliation, but they do not have any relevant impact on survival, the prognosis being poor, with an estimated overall survival less than 1 year from diagnosis.

## Background

Breast metastases (BMs) from extramammary tumors are extremely rare [1–6]. Frequencies of 0.5% and 6.6% have been reported in clinical and autopsy studies, respectively, the most common primary tumors being contralateral breast carcinoma followed by lung, gynecological, gastrointestinal, melanoma, and hematological cancers [1–6].

BMs are very difficult to distinguish clinically and radiologically from primary, benign or malignant breast lesions [6–9]. Histological and immunohistochemical studies are necessary for an accurate pathologic diagnosis [10–12]. BM treatment is extremely complex and depends on multiple factors, such as histology, the patient’s clinical condition and comorbidities, the presence of concomitant extramammary metastases, lymph node status, and the interval from primary tumor diagnosis [6–8].

Because BM is usually indicative of disseminated disease, its prognosis is poor, with an estimated overall survival less than 1 year from diagnosis [6–8]. BM from squamous cell head and neck carcinoma is very uncommon, with only a few cases reported in the literature to date [13, 14]. In our case, tumor relapse was clinically aggressive and led to patient's death 1 month after BM diagnosis, before starting any antitumoral treatment.

## Case presentation

In May 2016, a 47-year-old Caucasian woman was hospitalized for pain in the left knee resistant to common analgesics. She presented with a past history of epilepsy and gastroesophageal reflux disease. She was a hairdresser, and she smoked more than 20 cigarettes/day. She was married, and she had three healthy children. She had no family history of malignancy. She did not take any particular medication regularly. In 2014, she had undergone radical resection of a squamous cell carcinoma of the oropharynx (stage pT2, pN1), then received adjuvant radiotherapy. Her follow-up was uneventful until April 2016, when multiple bone metastases from a squamous cell carcinoma were histologically confirmed.

The patient’s oxygen saturation on admission was 96%, and her blood pressure and heart rate were normal at 124/82 mmHg and 95 beats/minute, respectively. Her oral temperature was 36.8 °C. The result of her physical examination was normal except for a bulky, painful lesion of the left knee and a solitary, painless, intramammary lesion of 4 cm in the right upper external quadrant (UEQ), without any skin retraction, associated with multiple fixed right axillary and supraclavicular lymph nodes.

The patient’s pH was 7.43, her partial pressure of arterial oxygen was 72 mmHg, and her partial pressure of arterial carbon dioxide was 36 mmHg. Laboratory tests revealed normocytic normochromic anemia (8.6 g/dl), hypoalbuminemia (26 g/dl), and severe hypercalcemia (3.31 mmol/L, ionized calcium 1.99 mmol/L). The patient’s renal and hepatic function was normal.

**Fig. 1:**
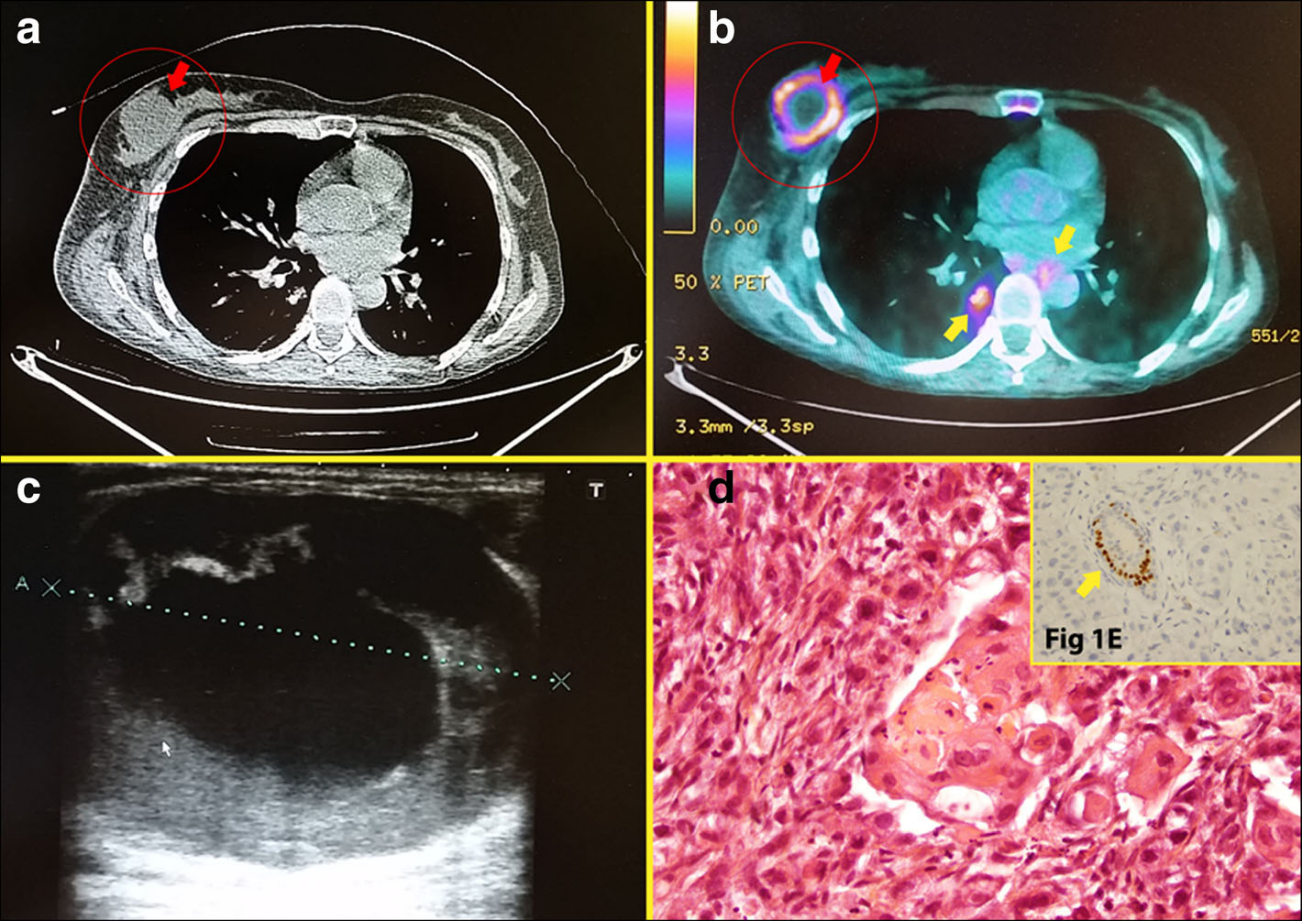
**a** Chest computed tomographic scan showing an irregular tumoral lesion of the right breast (*circled red arrow*). **b** Positron emission tomographic scan showing a hypermetabolic intramammary lesion with central necrosis (*circled red arrow*) associated with multiple mediastinal hypermetabolic lymph nodes (*yellow arrows*). **c** Breast ultrasound showing a hypoechogenic tumor lesion of 4-cm diameter in the upper external quadrant of the right breast without any spiculation. **d** Histological specimen (hematoxylin and eosin (H&E) stain, original magnification ×40) showing breast parenchyma massively infiltrated by medium-sized tumor cells with an eosinophilic cytoplasm and an anisokaryotic and hyperchromatic nucleus. The signs of mitosis are moderately frequent. Tumor cells are arranged in massifs or compact and, more rarely, cribriform spans within an abundant fibroinflammatory stroma. Focal areas of Malpighian inflection are observable without any *in situ* component. **e** Immunohistochemistry revealing tumor cells that are negative for estrogenic hormone receptors in contrast with normal canal breast cells, which regularly express these receptors (*yellow arrow*)

Whole-body computed tomography revealed multiple lymph node, peritoneal, splenic, lung, and bone metastases, as well as a solitary breast lesion in the right UEQ (Fig. 1a). A positron emission tomographic scan documented multiple visceral lymph nodes and bone metastases with a single hypermetabolic lesion of the right breast (Fig. 1b). Breast ultrasound confirmed the presence of a hypoechogenic tumor lesion of 4 cm in the UEQ that was associated with multiple axillary and infra-/supraclavicular adenopathies (Fig. 1c).

A percutaneous echo-guided biopsy of the breast lesion was performed. Histology revealed well- to moderately differentiated squamous tumor cells infiltrating the breast tissue without any *in situ* ductal or lobular component or desmoplastic reaction (Fig. 1d). Immunohistochemistry showed that the tumor cells were positive for anti-p40 and anti-p63 and negative for cytokeratins 7 and 20, hormone receptors, and human epidermal growth factor receptor 2, confirming the diagnosis of a BM from a squamous cell carcinoma (Fig. 1e).

Analgesic palliative radiotherapy was performed at the tibial bone metastasis. The patient’s hypercalcemia normalized after intravenous hydration and zoledronic acid administration. Her anemia was treated with a blood transfusion of 2 U of red cell concentrate. Considering the quick worsening of the patient’s clinical condition, best supportive care was initiated, and the patient died of acute respiratory insufficiency in June 2016 before any systemic antitumoral treatment was started.

## Discussion

We report a case of a 47-year-old Caucasian woman who presented to our hospital with a solitary BM in the context of multiple tumor diffusion of a squamous cell carcinoma of the oropharynx radically treated 2 years before with surgery and adjuvant radiotherapy. Compared with other cases reported in the literature, in our patient, tumor relapse showed very aggressive clinical behavior, leading to the patient’s death 1 month after BM diagnosis and before any antitumoral treatment was started.

BMs from extramammary tumors are extremely rare, the most common primary tumors being contralateral breast carcinoma followed by lung, gynecological, gastrointestinal, melanoma, and hematological cancers [1–6]. BMs are more common in women (92.2%), in the left breast (46%), and in the UEQ. They are bilateral in only 13.7% of cases [1–6].

The mean age at diagnosis is 50 years (range 32–87 years). BMs are often metachronous, and they appear, on average, 30 months after diagnosis of the primary extramammary malignancy. BM is the first sign of the primary extramammary tumor in 51% of cases [1–6].

Several hypotheses have been postulated to explain BM physiopathogenesis, the two most popular being the “seed and soil” theory and breast vascularization that is more important in the UEQ, supporting clinical and epidemiological evidence of the high BM frequency in this site [6].

Clinically, BMs present as round, rapidly growing, painless, and mobile masses, without any skin dimpling, nipple retraction, or bloody nipple discharge owing to their extraductal development [1–6]. On mammograms, BMs typically appear as well-circumscribed lesions without spiculations, thickening of the skin, or peritumoral stromal reaction, but they can be mistaken for benign (fibroadenoma) as well as primary breast malignancies [1–6]. Microcalcifications are unusual, and they have been reported in only a few cases of ovarian carcinoma with psammoma bodies [6–9]. At the ultrasound examination, hypoechogenic nodules with indistinct and irregular margins are often seen with or without penetrating vascularity, this latter being very suggestive of malignancy [6–9].

In a recent review, typical ultrasound features of BMs included single or multiple round to oval-shaped, well-circumscribed hypoechogenic masses without spiculations, calcifications, or architectural distortion [6–9]. However, lesions show variable radiological features in some cases, and the possibility of a BM should be suspected for a breast tumor in a patient with a history of cancer, even if clinically or radiologically benign [6–9].

Because it is very difficult to distinguish BMs clinically and radiologically from other primary breast tumors, histology plays a pivotal role in accurate diagnosis, which is essential to tailor an appropriate treatment [6–8]. Core percutaneous biopsy is comparatively better than fine-needle aspiration biopsy because histology and immunohistochemistry are often necessary for a differential diagnosis [9–12].

Histologically, BMs show a periductal and perilobular location, lack of any *in situ* ductal or lobular component, absence of a desmoplastic reaction and elastosis due to their fast growth, a sharp transition at the border of the tumor, and the presence of subcutaneous tissue infiltration [6, 9–12]. Also, because most primary breast carcinomas originate in the ducts or lobules of the breast, the finding of *in situ* (intraductal) carcinoma is more supportive of a primary breast tumor [9–12]. However, when a well-circumscribed breast tumoral lesion is identified showing lack of *in situ* components, the possibility of a BM should be considered and excluded, especially in high-grade and hormone receptor-negative tumors [9–12].

In a recent retrospective analysis by Buisman *et al.*, the diagnosis had to be corrected postoperatively in four patients (9%), supporting the point that BM diagnosis can be very difficult not only because clinical presentation may be similar to primary breast malignancies but also because it may be the first presentation of an unknown metastatic disease [9]. In addition, the diagnosis is hard to make on the basis of cytology alone. Obviously, proper clinical information may be helpful and should be provided to the pathologist.

Management of BM patients is extremely complex and depends on multiple factors, such as the patient’s clinical condition and comorbidities, the presence of concomitant extramammary metastases, histology, lymph node status, and the interval from primary tumor diagnosis [6]. Because BMs are usually associated with other concomitant extramammary metastases, supporting the evidence of an aggressive disseminated disease, surgery is indicated only for symptom palliation, such as in cases of local disease involving the skin, areola, or nipple [1–6] or when an isolated, metachronous BM is seen with a long interval from the primary tumor diagnosis. A simple mastectomy may be the treatment of choice in cases of large, ulcerated, or deep lesions causing severe pain or hemorrhage [1–6].

Instead, systemic therapy is required for most of these patients [1–6]. Using a combination of local therapy with systemic chemotherapy may also be considered if the patient has ulceration of the breast mass or invasion of the chest wall, as well as disseminated metastases [1–6]. The prognosis for patients with BMs is very poor, with an estimated overall survival less than 1 year from diagnosis [1–6].

## Conclusions

Our patient presented with an isolated BM deriving from a squamous cell carcinoma of the oropharynx treated 2 years before in the context of a disseminated and very aggressive disease that did not allow any antitumoral treatment. BM is rare and often clinically and radiologically misdiagnosed. It should be considered in any patient with a cancer history and confirmed by histology and immunohistochemistry. BM treatment has to be carefully tailored, taking multiple clinical and tumoral factors into consideration. The particularity of this case relies on the rarity of BM arising from squamous cell head and neck carcinoma, with only a few cases reported in the literature to date, as well as on the very aggressive clinical behavior of the disease, leading to the patient’s death 1 month after her BM diagnosis and before any systemic treatment was started.

## Abbreviations

BM: Breast metastasis
UEQ: Upper external quadrant
